# Supplementary material for: Genome-Wide Analysis of the Polygalacturonase Gene Family in Macadamia and Identification of Members Involved in Fruit Abscission
Source: Plants (Basel). 2025 May 25;14(11):1610. doi: 10.3390/plants14111610 (PMC12157947; doi:10.3390/plants14111610)
Supplement: Supplementary file 1 [file plants-14-01610-s001.zip › Supplementary Figures.pdf]

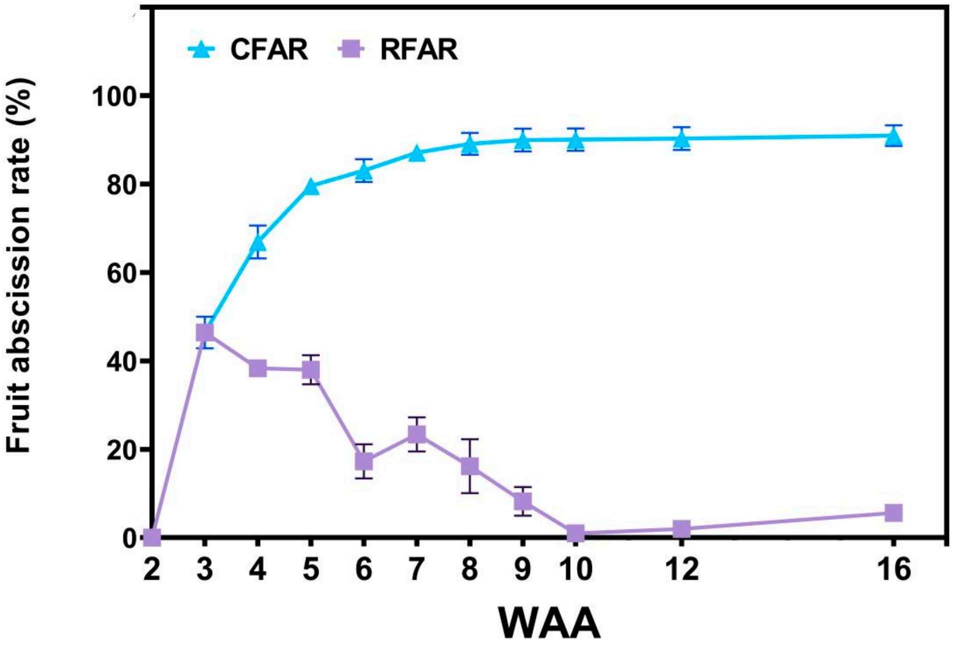

Figure S1. Temporal dynamics of macadamia fruit abscission. WAA: weeks after anthesis; CFAR: cumulative fruit abscission rate; and RFAR: relative fruit abscission rate. Each value represents the mean of four biological replicates, with the standard error indicated by the vertical bar.

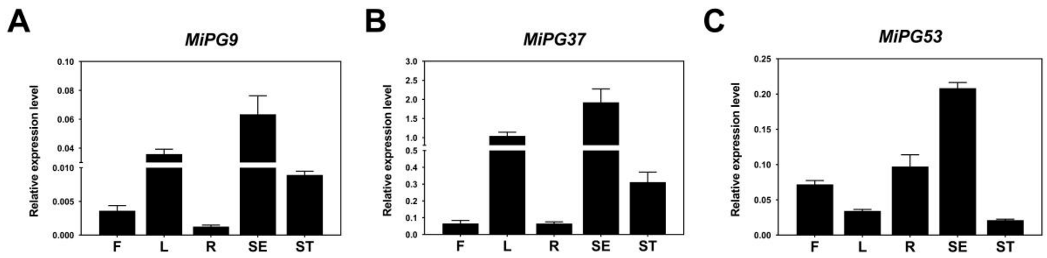

Figure S2. Tissue-specific expression profiles of *MiPG9*, *MiPG37*, and *MiPG53* in macadamia. F: flowers; L: leaves; R: roots; SE: seeds; and ST: stems. Each value represents the mean of three biological replicates, with the standard error indicated by the vertical bar.

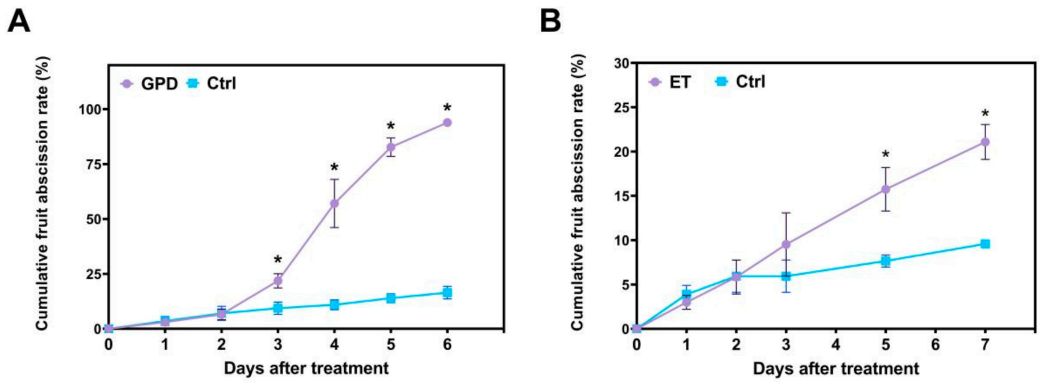

Figure S3. Effects of girdling with defoliation (A) and ethephon (B) treatments on the cumulative fruit abscission rate in macadamia. A purple circle represents girdling with defoliation (GPD)/ ethephon (ET) treatment, and a blue square represents the control (Ctrl). Each value represents the mean of three biological replicates, with the standard error indicated by the vertical bar. Significant differences at the 0.05 level according to the t-test are indicated with asterisks (*).
